# Phenolic-Rich Wild Edible Macrofungi: Antimicrobial Activity and Antioxidant Potential

**DOI:** 10.3390/molecules31060978

**Published:** 2026-03-14

**Authors:** Elif Ildız, Elif Yürümez Canpolat

**Affiliations:** Department of Biotechnology, Faculty of Science, Niğde Ömer Halisdemir University, Niğde 51240, Türkiye

**Keywords:** edible macrofungi, phenolic compounds, antioxidant activity, antimicrobial activity, multidrug-resistant bacteria, GC–MS, LC-–MS/MS

## Abstract

This study evaluated the antimicrobial and antioxidant activities and chemical properties of four wild edible macrofungi—*Tuber aestivum* (Wulfen) Spreng., *Terfezia claveryi* Chatin, *Agaricus arvensis* Schaeff. and *Bovistella utriformis* (Bull.) Demoulin & Rebriev—collected from different regions of Türkiye, with particular emphasis on the role of phenolic compounds. Methanol and hexane extracts were assessed for antimicrobial activity against Gram-positive, Gram-negative, multidrug-resistant (MDR) bacterial strains, and *Candida albicans* using minimum inhibitory concentration (MIC) assays. Total phenolic content (TPC) was determined, and antioxidant capacities were evaluated using DPPH (2,2-diphenyl-1-picrylhydrazyl), ABTS (2,2′-azinobis-(3-ethylbenzothiazoline-6-sulfonic acid)), FRAP (ferric reducing antioxidant power), and CUPRAC (cupric ion reducing antioxidant capacity) assays. The chemical profiles of hexane extracts were characterized by GC–MS analysis, whereas methanol extracts were analyzed by LC–MS/MS. Methanol extracts with high content of phenolic compounds exhibited markedly higher antimicrobial activity than hexane extracts, especially against Gram-positive bacteria. *B. utriformis* and *A. arvensis* displayed the highest phenolic contents (29.61 ± 0.6 and 27.14 ± 0.59 mg GAE/g DW, respectively) and antioxidant activities, revealing a strong positive correlation between TPC and antioxidant capacity. LC–MS/MS analysis revealed catechin, cinnamic acid, and caffeic acid as prominent phenolic constituents, highlighting the role of polyphenols in the observed bioactivity. GC–MS profiling predominantly identified fatty acid methyl esters, particularly linoleic and oleic acids, together with minor phenolic derivatives, suggesting a possible synergistic interaction contributing to the overall biological potential. The results highlight phenolic-rich macrofungi as valuable natural sources of antioxidant and antimicrobial agents with potential applications.

## 1. Introduction

The emergence of antibiotic-resistant bacterial strains has become one of the most severe public health threats of the 21st century, as many infectious diseases that were once easily treated are becoming increasingly unmanageable [1,2]. This crisis is driven by the genetic ability of pathogens to acquire or mutate resistance genes through horizontal gene transfer, including processes such as conjugation, transformation, and transduction. The World Health Organization (WHO) identifies antimicrobial resistance as a worldwide challenge directly associated with high morbidity and mortality [3,4]. It is conservatively estimated that antibiotic resistance results in at least 23,000 deaths annually in the United States alone, with global predictions suggesting this figure could reach 10 million deaths per year by 2050. Furthermore, the pharmaceutical pipeline for new antibiotics has largely run dry, leaving a critical shortage of effective therapies and necessitating the development of novel antimicrobial alternatives [5].

Natural products have gained increasing attention as potential sources of novel bioactive compounds. Mushrooms are valuable organisms that have been used in both cuisine and traditional medicine since ancient times, due to their unique aroma and high nutritional value. Despite their low calorie and fat content, they are a rich source of proteins, vitamins, minerals, and dietary fiber [6,7,8]. Beyond these basic nutritional properties, mushrooms offer a wide range of health benefits through secondary metabolites such as phenolic compounds. The growing interest in mushroom polyphenols in the field of functional foods and nutraceuticals today stems from the potential of these bioactive compounds to prevent chronic diseases and improve human health [9,10,11]. The phenolic compounds found in mushrooms combat oxidative stress in a multifaceted manner by acting as both primary and secondary antioxidants [12]. These compounds break the chain reactions of auto-oxidation by neutralizing free radicals through hydrogen atom donation or electron transfer [13]. They protect cells from tissue damage or death by preventing DNA, RNA, protein, and lipid damage caused by reactive oxygen species (ROS) such as superoxide radicals, hydrogen peroxide, and hydroxyl radicals [14,15]. The antioxidant capacity of phenolics protects cells beyond their radical scavenging effect; it also defends cells by chelating ROS-producing metals (such as Fe and Cu) and inhibiting oxidase enzymes like NADH oxidase or lipoxygenase that lead to radical formation. Research confirms a direct and strong correlation between total phenolic content (TPC) in mushrooms and antioxidant capacity, with phenolic acids (gallic, caffeic, p-coumaric acid, etc.) being the main components responsible for this activity [12,16,17].

In addition to their antioxidant effects, mushroom polyphenols exhibit a broad spectrum of bioactivity. They have anti-inflammatory effects by suppressing the production of inflammatory mediators such as nitric oxide (NO) and reactive oxygen species [18,19,20]. Furthermore, they exhibit antitumor activity by inhibiting the proliferation of cancer cells and triggering apoptosis, while displaying antihyperglycemic properties by inhibiting α-glucosidase and α-amylase enzymes, thereby balancing blood sugar levels [21,22,23,24,25]. These compounds, which provide antimicrobial protection against microorganisms by inhibiting cell wall, protein, and nucleic acid synthesis, have also been scientifically documented to inhibit the tyrosinase enzyme responsible for melanin synthesis and to offer anti-osteoporotic effects that prevent bone loss [26,27]. Due to these multifaceted biological activities, fungal phenolics are recognized as valuable active compounds with applications in a wide range of fields.

Several genera included in the present study, including *Terfezia*, *Tuber*, *Agaricus*, and *Bovistella*, are known to contain phenolic compounds contributing to antioxidant and antimicrobial activities. While previous studies have reported bioactive properties of these genera, comparative evaluations integrating total phenolic content, antioxidant capacity, and antimicrobial activity of wild edible representatives collected from Türkiye remain scarce. The chemical diversity of wild edible fungi, particularly those collected from different ecological regions, represents an underexplored reservoir of bioactive molecules [28,29].

The application of fungus extracts against multidrug-resistant (MDR) bacterial strains has yielded promising results in recent clinical studies. Extracts from species such as *Pleurotus ostreatus*, *Fistulina hepatica*, and *Russula delica* have demonstrated potent inhibitory effects against high-priority pathogens, including methicillin-resistant *Staphylococcus aureus* (MRSA), *Escherichia coli*, and *Pseudomonas aeruginosa*. Specifically, methanolic extracts of the fungi, edible macrofungi in particular, were found to be more effective than conventional antibiotics against clinical isolates of vancomycin-resistant *S. aureus* and *Salmonella typhi*. These extracts contain a complex cocktail of molecules that can directly inhibit bacterial growth by targeting cell walls, mRNA, and protein synthesis [5,30,31]. Despite these promising findings, most previous studies have focused on single species or single extraction solvents, and comprehensive evaluations combining chemical profiling and activity assessment remain scarce.

In this context, the present study aimed to comparatively evaluate the chemical composition and biological activities of four species of wild edible fungi belonging to different families of Ascomycota and Basidiomycota collected from Türkiye between 2022 and 2023. Unlike previous reports focusing on single species or limited bioactivity screening, this study integrates (i) solvent-based extraction (methanol and hexane), (ii) determination of total phenolic content, (iii) assessment of antioxidant capacity, and (iv) determination of minimum inhibitory concentration (MIC) values against Gram-positive, Gram-negative, and multidrug-resistant bacterial strains.

## 2. Results and Discussion

### 2.1. Identification of the Macrofungi Samples

**Figure 1:**
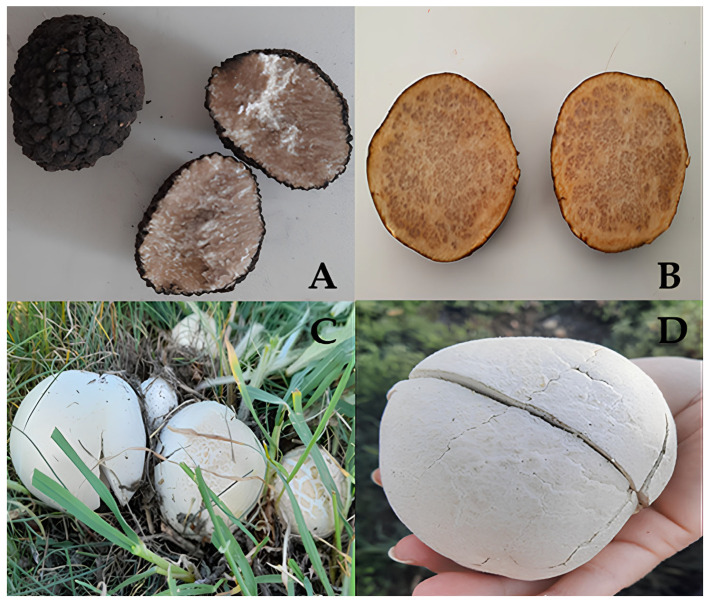
Macrofungi samples collected from field studies: (**A**) *Tuber aestivum*, (**B**) *Terfezia claveryi*, (**C**) *Agaricus arvensis*, and (**D**) *Bovistella utriformis*.

The four wild edible macrofungi collected during field studies were identified based on morphological characteristics according to the current literature [32,33,34]. Figure 1 shows the ascocarps and basidiocarps of the samples collected from their natural environment.

A further identification based on extraction, amplification and sequencing of the internal transcribed spacer (ITS) region of the rDNA, which is a highly conserved and widely used fragment for identification of the fungal samples due to its interspecific variations, was also performed to avoid possible confusion at the species level [35,36]. The sequence data was compared with the GenBank database via Basic Local Alignment Search Tool (BLAST) (nucleotide BLAST, accessed on https://blast.ncbi.nlm.nih.gov/Blast.cgi?PROGRAM=blastn&PAGE_TYPE=BlastSearch&LINK_LOC=blasthome), and the results revealed that the fungal samples were *Tuber aestivum* (Wulfen) Spreng. (1827), *Terfezia claveryi* Chatin (1892), *Agaricus arvensis* Schaeff. (1774) and *Bovistella utriformis* (Bull.) Demoulin & Rebriev (2017), which are in accordance with the morphological identification. The sequence data of the identified samples were also deposited in GenBank, and the accession numbers are listed in Table 1, which also indicate the GPS coordinates, locations, average altitudes and the collection dates of the samples.

### 2.2. Antioxidant Capability

One of the most important parameters in determining the bioactive potential of mushroom extracts is the total phenolic content (TPC) and the associated antioxidant activities. When these were examined, statistically significant differences were observed between species (*p* < 0.05). Based on the data presented in Table 2, the highest total phenolic content was detected in the *B. utriformis* sample with a value of 29.61 ± 0.6 mg GAE/g dry weight. This was followed by *A. arvensis* with 27.14 ± 0.59 mg GAE/g DW. In contrast, the hypogeous fungi (truffle group) *T. aestivum* (18.52 ± 1.06 mg GAE/g DW) and *T. claveryi* (16.66 ± 0.59 mg GAE/g DW) exhibited lower phenolic content.

These findings are consistent with recent studies in the literature. Several studies have reported that members of Basidiomycota (especially members of the Agaricaceae and Lycoperdaceae families) generally have a higher secondary metabolite synthesis capacity compared to hypogeous fungi [37,38]. The high phenolic content of *B. utriformis* supports the scientific basis for the use of this species and its close relatives (*Calvatia* sp.) as wound healers in traditional medicine. The results of antioxidant activity tests (DPPH, ABTS, FRAP, and CUPRAC) showed a strong positive correlation with total phenolic content. *B. utriformis* and *A. arvensis* exhibited the highest activity in all tested methods.

In particular, *B. utriformis* and *A. arvensis* performed higher free radical scavenging activity for the DPPH assay with 4.93 ± 0.04 and 4.92 ± 0.01 mmol TE/g DW, respectively. On the other hand, their ABTS^+^ cation-degrading values were 1.66 ± 0.02 and 1.90 ± 0.05 mmol TE/g DW, respectively. For both antioxidant assays investigated with free radicals, *T. aestivum* and *T. claveryi* exhibited lower scavenging activities. The FRAP test, which measures metal chelation and reduction capacity, yielded notable values for *A. arvensis* with 11.16 ± 0.27 mmol AAE/g DW, and the CUPRAC test yielded notable values for *B. utriformis* with 10.45 ± 0.39 mmol TE/g DW, while *T. aestivum* and *T. claveryi* exhibited lower values again for both assays.

**Figure 2:**
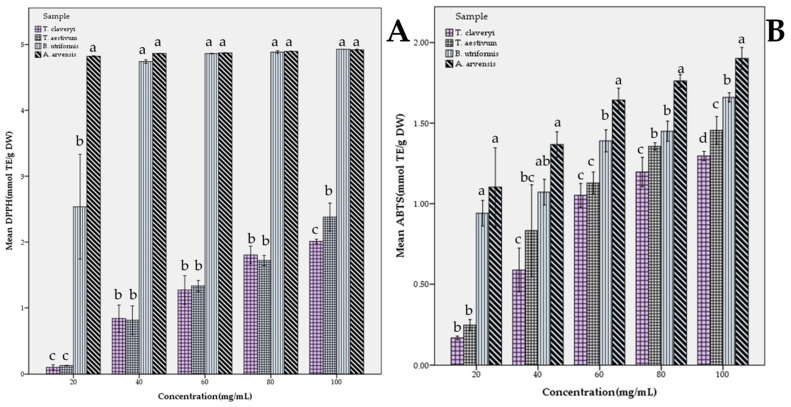
Dose-dependent free radical scavenging activity of fungal extracts: (**A**) DPPH radical scavenging activity and (**B**) ABTS^+^ cation-degrading activity. Different lowercase letters at each concentration indicate a significant difference at *p* < 0.05.

In Figure 2, the DPPH and ABTS free radical scavenging activities of the fungal samples were demonstrated according to the extract concentrations. All samples exhibited an increasing antioxidant activity with regard to the increasing extract concentration. Interestingly, for DPPH scavenging activity, it was observed that the extract of *A. arvensis* reached a plateau at the lowest dose of 20 mg/mL, exhibiting values close to the antioxidant activity values displayed at the highest concentration. Another noteworthy point is that no significant difference was observed between the DPPH scavenging effects of *A. arvensis* and *B. utriformis* extracts at concentrations of 40 mg/mL and above. On the other hand, a significant difference was clearly observed at all concentrations between *T. claveryi* and *T. aestivum* and these two fungi. In terms of ABTS cation-degrading activity, a statistically significant difference (*p* < 0.05) was found between *A. arvensis* and all other fungi at all concentrations tested. In the study conducted with six wild edible fungi, a 10.92 ± 0.60 mg TE/g extract DPPH scavenging and 51.91 ± 2.72 mg TE/g extract ABTS degrading value were reported for *Lycoperdon utriforme* (*syn. B. utriformis*) methanol extract [39], which supports the findings of this study. Similarly, the DPPH radical scavenging value of *A. arvensis* collected from nature was reported as 65.73% for 20 mg/mL extract [40], while the EC_50_ ABTS^+^ degrading activity of this fungus was reported as 3.19 ± 0.3 mg/mL [41].

**Figure 3:**
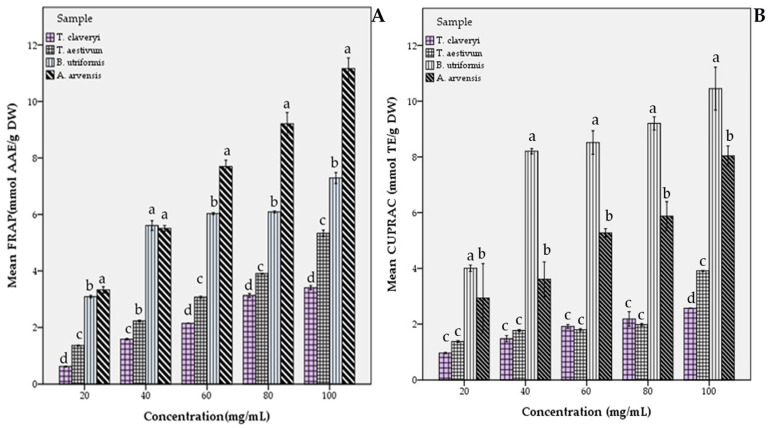
Dose-dependent metal ion reducing capability of fungal extracts: (**A**) ferric reducing antioxidant power (FRAP) and (**B**) cupric reducing antioxidant capacity (CUPRAC). Different lowercase letters at each concentration indicate a significant difference at *p* < 0.05.

Similar to the free radical scavenging activity assays, metal ion reducing capacity assays (FRAP and CUPRAC) revealed that all samples demonstrated an increasing pattern of antioxidant capacity with the increase in extract concentration (Figure 3). In the FRAP test, *A. arvensis* showed a significant difference at the *p* < 0.05 level at all tested concentrations compared to the other fungal samples investigated. This is also the case for *B. utriformis* in the CUPRAC test. For all concentrations tested, a significant difference was found compared to the other samples.

In a study on *T. claveryi*, the aqueous extract exhibited markedly higher total phenolic (176.67 mg GAE/g DW) content compared to other solvent extracts, and this value was positively correlated with antioxidant activity [42]. The authors further emphasized that antioxidant and antibacterial activities were strongly solvent-dependent, with aqueous and methanolic extracts showing different bioactivity profiles. In contrast, the present study employed methanolic extracts and revealed substantially lower TPC and antioxidant activities for *T. claveryi*, which is consistent with the solvent effect reported in the literature. The lower FRAP and CUPRAC values observed in methanolic *T. claveryi* extract likely reflect a reduced extraction efficiency of hydrophilic phenolic compounds, which dominate the antioxidant profile of this species. Therefore, the discrepancy between the high bioactivity reported for aqueous extracts of *T. claveryi* and the comparatively lower antioxidant performance observed in the present study can be directly attributed to differences in the extraction solvent rather than species-specific limitations.

In the present study, the methanolic extract of *Tuber aestivum* exhibited lower total phenolic content and antioxidant activity compared to epigeous Basidiomycota species (*B. utriformis* and *A. arvensis*). This finding is consistent with the literature reporting the relatively limited antioxidant potential of *T. aestivum*. In a comprehensive study conducted by Beara et al. [43], the total phenolic content of methanolic and aqueous extracts of *T. aestivum* was reported to range between 11.72 and 18.68 mg GAE/g dry weight, while the antioxidant activities assessed by DPPH, FRAP, and lipid peroxidation inhibition assays were found to be at moderate levels. Although the total phenolic content values obtained for *T. aestivum* in the present study are consistent with this range, the FRAP capacity reported in the same study (maximum 13.4 mg AAE/g dry weight) is noticeably lower than the FRAP and CUPRAC values determined for *A. arvensis* and *B. utriformis*. Beara et al. [43] emphasized that the limited antioxidant activity of *T. aestivum* may be associated with its hypogeous growth habit, lower phenolic density, and the restricted structural diversity of phenolic compounds.

In a previous study evaluating the antioxidant properties of *Lycoperdon utriforme*, high total antioxidant capacity (203.61 mg TE/g extract) and notable FRAP (10.85 mg TE/g extract) and CUPRAC (39.66 mg TE/g extract) values were reported. When these findings are compared with the present study, *B. utriformis* (*syn. Lycoperdon utriforme*) exhibited comparable reducing power in the FRAP assay, with a value of 11.159 ± 0.27 mmol AAE/g DW, indicating a similar metal-reducing capacity despite differences in extraction protocol and expression units. However, CUPRAC activity in the mentioned study was considerably higher, which may be attributed to the differences in solvent polarity and extraction efficiency [39]. In the present study, methanolic extracts were used, whereas the solvent type was not standardized across studies, which is known to strongly influence the recovery of redox-active compounds. Nevertheless, the consistent observation of strong FRAP responses in both studies supports the conclusion that *B. utriformis* possesses an inherently high electron-donating and metal-reducing antioxidant potential.

In the study evaluating phenolic profiles and antioxidant properties of several *Agaricus* species, *Agaricus arvensis* was reported to exhibit one of the highest DPPH radical scavenging activities within the genus, ranking second after *A. bitorquis* and exceeding commonly consumed species such as *A. bisporus* and *A. campestris*. This finding indicates a relatively strong antiradical capacity for *A. arvensis* based on DPPH assay results [10]. In agreement with these findings, the present study demonstrated that *A. arvensis* showed high antioxidant activity not only in DPPH but also consistently across ABTS, FRAP, and CUPRAC assays. Notably, the FRAP value obtained for *A. arvensis* methanol extract with 100 mg/mL concentration in the present study (11.16 ± 0.27 mmol AAE/g DW) confirms its strong reducing capacity, extending the single-assay DPPH results reported previously to multiple antioxidant mechanisms. Therefore, while the mentioned study characterized *A. arvensis* primarily by its DPPH scavenging ability, the current results demonstrate that this species exhibits a broad and robust antioxidant profile across different assay systems.

Phenolic compounds are known to form a protective shield against oxidative stress due to their ability to stabilize free radicals (DPPH and ABTS cations) and reduce metals (FRAP/CUPRAC). The data obtained in our study reveal that Basidiomycota samples have stronger free radical scavenging potential compared to the investigated Ascomycota members. In agreement with the present findings, several studies have shown that phenolic compounds in mushrooms are closely linked to antioxidant activities across multiple assay systems. LC-MS/MS profiling of edible and wild mushrooms has identified various phenolic acids and flavonoids, such as catechin, caffeic acid, and ferulic acid, which correlate with strong antioxidant potential [29,44]. Our LC–MS results confirmed the presence of these compounds in *Tuber aestivum*, *Terfezia claveryi*, *Agaricus arvensis*, and *Bovistella utriformis*, showing that phenolic content underpins the DPPH, ABTS, FRAP, and CUPRAC antioxidant activities observed. Furthermore, GC–MS analysis of the same mushroom samples revealed a diverse profile of fatty acids, including both saturated and unsaturated species, which are also known to contribute to antioxidant and health-promoting properties. The integration of LC–MS and GC–MS data indicates that the antioxidant capacity of these mushrooms arises not only from phenolic compounds but also from fatty acids, highlighting the multi-component nature of their bioactive potential.

### 2.3. Chemical Compositions of the Fungal Samples

LC–MS analysis was conducted to determine the concentrations of selected phenolic compounds in *T. aestivum*, *T. claveryi*, *A. arvensis*, and *B. utriformis*. Phenolic compounds were detected using LC–MS/MS in Multiple Reaction Monitoring (MRM) mode. Precursor and product ions, ionization modes, and retention times are shown in Table 3. Catechin hydrate and cinnamic acid were detected in positive ion mode, while other compounds were analyzed in negative ion mode. Catechin was detected in all species, reaching the highest concentrations in *B. utriformis* (18.657 μg/g DW) and *A. arvensis* (16.359 μg/g DW). Cinnamic acid was only detected in epigeous species, with concentrations of 65.178 μg/g DW in *A. arvensis* and 69.981 μg/g DW in *B. utriformis*; it was not detected in hypogeous species (*T. aestivum* and *T. claveryi*). Caffeic acid showed the highest levels in *T. aestivum* (0.952 μg/g DW) and *T. claveryi* (0.804 μg/g DW), while it was present at very low levels in *A. arvensis* and *B. utriformis*. 2,5-Dihydroxybenzoic acid was only detected in *T. aestivum* at 0.041 μg/g DW. Trans-ferulic acid was detected at low concentrations in all four species, with the highest level observed in *T. aestivum* (0.098 μg/g DW). Among flavonoids, quercetin was detected at low levels only in hypogeous species, while luteolin was found exclusively in *T. aestivum* at a very low concentration (0.002 μg/g dw). Myricetin, naringenin, chrysin, tannic acid, and ellagic acid were not detected in any of the species. Overall, hypogeous truffle species (*T. aestivum* and *T. claveryi*) exhibited low to moderate phenolic profiles in terms of catechin and caffeic acid, whereas epigeous species (*A. arvensis* and *B. utriformis*) showed higher concentrations of catechin and cinnamic acid. These results indicate species-specific metabolic differences and habitat-related phenolic diversity. These findings are in agreement with previous reports indicating the presence of various phenolic acids and flavonoids, such as gallic acid, protocatechuic acid, hydroxycinnamic acid derivatives, catechin, and myricetin [45,46].

In the study by [10], *A. arvensis* was reported to contain phenolic acids including gallic, caffeic, and ferulic acids, while flavonoids were generally not detected; total phenolic content was correlated with high antioxidant activity. The results of the present study are consistent with these findings regarding the predominance of phenolic acids and the contribution of catechin and cinnamic acid to antioxidant potential. The detection of catechin in both hypogeous and epigeous species in our study further supports the role of flavan-3-ols in contributing to antioxidant potential. Dundar et al. [40] investigated methanol extracts of seven mushroom species from Türkiye and found significant antioxidant activity, along with the presence of phenolic acids including protocatechuic, caffeic, syringic, and p-coumaric acids. Çayan et al. [47] analyzed 26 mushroom species for their phenolic acid content and reported a total of 16 phenolic and organic acid compounds. The majority of the samples contained gallic acid, fumaric acid, barrosprotocatechuic acid, catechin hydrate, and trans-cinnamic acid. Barros et al. [48] examined sixteen wild Portuguese mushroom species and identified phenolic acids as the predominant compounds. They also reported that *Lycoperdon* species *(L. molle* and *L. perlatum*, currently classified as *Bovistella*) exhibited the highest total phenolic contents, which is consistent with the results obtained in our study.

The GC-MS analysis of hexane extracts from *Tuber aestivum*, *Terfezia claveryi*, *Agaricus arvensis*, and *Bovistella utriformis* revealed a complex chemical profile composed primarily of fatty acid esters, along with a diverse array of alkanes, alcohols, ketones, and phenols. Fatty acid esters constituted the most abundant class of compounds across all four species, though their specific distributions varied significantly. *T. claveryi* was characterized by a high concentration of hexadecanoic acid methyl ester (26.58%), whereas *T. aestivum* was dominated by 9,12-octadecadienoic acid methyl ester (19.9%). In contrast, the profiles of *A. arvensis* and *B. utriformis* were distinctively rich in octadecanoic acid methyl ester, which accounted for 22.78% and 26.18% of their relative concentrations, respectively. Notably, the unsaturated fatty acid ester 9-octadecenoic acid methyl ester acted as a distinguishing marker between the groups; it was prominent in the truffle species (*T. aestivum* and *T. claveryi*, >11%) but present only in trace amounts in *A. arvensis* and *B. utriformis* (~1%).

**Figure 4:**
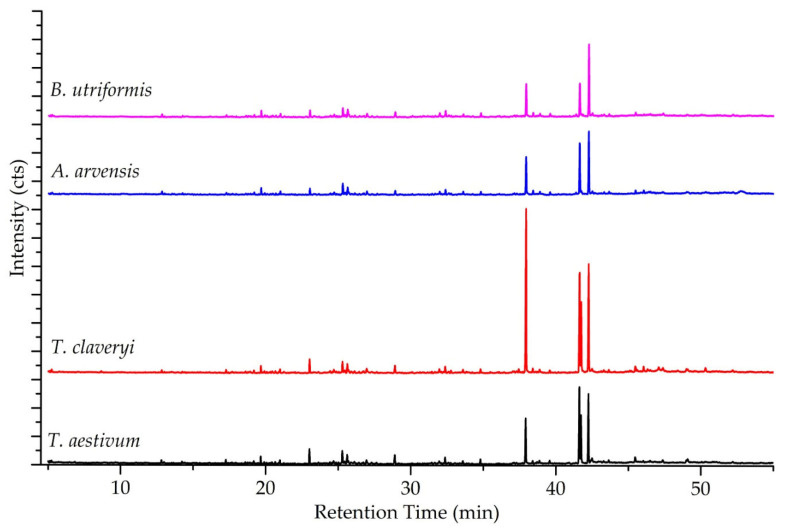
The GC-MS chromatograms of hexane extracts obtained from the studied mushroom species.

Beyond the dominant fatty acid esters, the extracts contained various hydrocarbons and volatile organic compounds. Alkanes represented the most chemically diverse group, with compounds such as tetradecane, hexadecane, and octadecane detected across the species, generally ranging from trace amounts to approximately 5%. *B. utriformis*, in particular, showed a slightly higher accumulation of specific long-chain alkanes like octadecane (4.99%) and hexadecane (4.16%). Other bioactive constituents included ketones, specifically 2,6-di-t-butyl-4-methylene-2,5-cyclohexadiene-1-one, found in all samples (1.75–4.02%), and minor concentrations of phenolic compounds and alcohols (Table 4). Overall, while the compositions were generally similar, the variations in relative concentrations, especially within the fatty acid ester profiles, highlighted distinct chemical differences between the investigated Ascomycota and Basidiomycota species (Figure 4).

Several studies have reported that the lipid fraction of mushrooms and truffles is dominated by long-chain fatty acids, particularly oleic and linoleic acids, usually detected as methyl esters in GC–MS analyses. Kalač [49] comprehensively reviewed mushroom lipid composition and demonstrated that linoleic acid is the predominant unsaturated fatty acid in most edible mushrooms and truffles, followed by oleic acid. Similarly, Reis et al. [50] reported that methanolic extracts of wild mushrooms were characterized by high proportions of unsaturated fatty acids, with linoleic acid frequently accounting for more than 50% of total fatty acids.

In agreement with these reports, the present study revealed that all four investigated mushroom species (*T. aestivum*, *T. claveryi*, *A. arvensis*, and *B. utriformis*) contained substantial amounts of linoleic and oleic acid methyl esters, confirming the predominance of unsaturated fatty acids. Comparable fatty acid patterns have also been described for truffle species by Tejedor-Calvo et al. [51], who reported that *Tuber* spp. exhibit lipid profiles rich in unsaturated fatty acids, contributing to both their nutritional quality and aroma characteristics. The fatty acid profiles reported in this study are in agreement with previously published data for truffle species. In a chemical characterization of multiple *Tuber* and *Terfezia* truffles, oleic and linoleic acids were identified as the predominant fatty acids, with unsaturated fatty acids constituting a major fraction of the total lipid content (38.2–79.3%) in most species studied.

The fatty acid profile obtained for *B. utriformis* in the present study shows a strong similarity with previously reported data for *Lycoperdon utriformis* (*syn. Bovistella utriformis*). In both species, long-chain C16–C18 fatty acids constitute the dominant lipid fraction, with palmitic, stearic, oleic and linoleic acids being the major components. The predominance of unsaturated fatty acids over saturated ones reported for *L. utriformis* is consistent with the high relative abundance of oleic and linoleic acid methyl esters detected in *B. utriformis* by GC–MS analysis. Although differences in relative percentages were observed, these variations can be attributed to differences in geographical origin, extraction solvent and analytical methodology. Overall, the comparable lipid patterns support the notion that members of the Lycoperdaceae family share conserved fatty acid biosynthetic characteristics. Similarly, in the present GC–MS analysis, linoleic acid, oleic acid, stearic acid and palmitic acid methyl esters were among the most abundant lipid-derived constituents across the four mushroom species. This concordance with the literature not only reinforces the reproducibility of fatty acid distribution patterns in truffles and related fungi but also provides a solid chemical basis for interpreting biological activities linked to lipid composition in these taxa.

### 2.4. Antimicrobial Activity

The antimicrobial activities of methanol and hexane extracts obtained from *T. aestivum*, *T. claveryi*, *A. arvensis*, and *B. utriformis* were evaluated against a panel of Gram-positive (+), Gram-negative (−), and multidrug-resistant (MDR) bacterial strains and *Candida albicans*. The minimum inhibitory concentration (MIC) values are presented in Table 5 and Table 6. Overall, methanol extracts exhibited substantially stronger antimicrobial activity compared to hexane extracts. Among the tested fungi, *T. claveryi* and *B. utriformis* methanol extracts demonstrated the broadest and most potent inhibitory effects. The lowest MIC values were observed against *E. faecalis* ATCC 29212, with MICs of 0.8 mg/mL for *T. aestivum*, *T. claveryi*, and *B. utriformis*, and 1.6 mg/mL for *A. arvensis*. Similarly, *B. cereus* RSKK 863 and *B. subtilis* DSMZ 1971 showed high susceptibility to methanol extracts, particularly those of *T. aestivum* and *T. claveryi* (MICs ranging from 1.6 to 3.125 mg/mL). Notably, the methanol extracts exhibited variable inhibitory activity against multidrug-resistant (MDR) strains. The methanol extract of *A. arvensis* showed the lowest MIC value against *S. pneumoniae* MDR (12.5 mg/mL) and moderate activity against *E. coli* MDR and *K. pneumoniae* MDR (25 mg/mL). In contrast, the methanol extract of *T. claveryi* exhibited inhibitory activity against *K. pneumoniae* MDR and methicillin-resistant *S. aureus* (MRSA), with MIC values of 25 mg/mL. The methanol extract of *B. utriformis* showed activity against *S. pneumoniae* MDR and MRSA with MIC values of 25 and 50 mg/mL, respectively, whereas the methanol extract of *T. aestivum* did not exhibit inhibitory activity against MRSA and showed higher MIC values against other MDR strains.

Hexane extracts exhibited limited antimicrobial activity overall. Inhibitory effects were mainly restricted to certain Gram-positive bacteria, particularly *Bacillus* species and *M. luteus* M41, with MIC values generally ranging from 25 to 100 mg/mL. Most Gram-negative bacteria, MDR strains, and *C. albicans* showed no detectable inhibition in response to hexane extracts. In the hexane extracts, inhibitory activity was detected only against a limited number of bacterial strains, including *E. faecalis*, *B. subtilis*, *M. luteus*, *S. epidermidis*, *K. pneumoniae* MDR, and *B. cereus*, with MIC values ranging from 25 to 200 mg/mL, whereas no inhibition was observed for several other tested strains, including *E. coli* ATCC 25922 and *E. aerogenes* ATCC 13048.

In the present study, the antimicrobial potential of hexane and methanol extracts obtained from four wild edible fungal species was systematically evaluated against a broad spectrum of microorganisms, including Gram-positive, Gram-negative, multidrug-resistant (MDR) bacteria, and *C. albicans*. The results clearly demonstrated that methanol extracts exhibited significantly higher antimicrobial activity than hexane extracts, suggesting that bioactive compounds extracted by polar solvents play a major role in the observed inhibitory effects. The superior activity of methanol extracts is consistent with previous studies reporting that phenolic compounds, flavonoids, organic acids, and other secondary metabolites extracted using polar solvents are primarily responsible for antimicrobial effects in fungi [52,53]. In particular, truffles and other macrofungi are known to be rich in phenolic acids and bioactive peptides, which can disrupt microbial cell membranes and interfere with metabolic pathways [54]. These results are supported by recent studies showing that mushroom extracts often display higher antimicrobial activity against Gram-positive bacteria than Gram-negative bacteria, likely due to structural differences in the bacterial cell envelope that limit antimicrobial penetration in Gram-negative organisms.

Previous studies have demonstrated that *Terfezia* species possess notable antimicrobial potential, although the magnitude of activity varies considerably depending on the extraction solvent and tested microorganisms [42,55,56,57]. For instance, Doğan and Aydın reported strong antimicrobial activity of *Terfezia boudieri*, particularly with acetone and chloroform extracts, showing low MIC values (<100 μg/mL) against both Gram-positive and Gram-negative bacteria as well as *Candida albicans*. In contrast, methanol extracts in that study generally exhibited moderate activity, with MIC values ranging between 156 and 625 μg/mL. In the present study, methanol extracts of *T. claveryi* and *T. aestivum* exhibited measurable antimicrobial activity against a broad spectrum of bacteria, including MDR strains, although at higher MIC values (mg/mL range) compared to those reported by Doğan and Aydın [58]. This difference is likely attributable to variations in extraction protocols, solvent polarity, fungal species, and test strains. Notably, the antifungal activity observed against *Candida albicans* in the present study, particularly for *T. claveryi* (MIC: 3.125 mg/mL), aligns with earlier findings demonstrating the susceptibility of *Candida* species to *Terfezia* extracts.

In line with the findings of the present study, previous research has also demonstrated the antimicrobial potential of *Handkea utriformis* (*syn*. *Bovistella utriformis*). Petrović et al. [59] reported that the methanol extract of *B. utriformis* exhibited inhibitory activity at varying concentrations against a range of clinically relevant microorganisms, including *S. aureus*, *E. faecalis*, *P. aeruginosa*, *E. coli*, and *C. albicans.* The consistency between these findings and our results further supports the notion that methanol is an effective solvent for extracting bioactive compounds from *B. utriformis* and highlights this species as a promising source of antimicrobial agents with broad-spectrum activity. Sevindik et al. [60] reported that the methanol extract of *B. utriformis* exhibited notable antimicrobial activity against *E. coli* at a concentration of 200 μg/mL. These findings are in agreement with the results of the present study, in which the methanol extract of *B. utriformis* demonstrated inhibitory activity against *E. coli*, although higher MIC values were observed. The differences in effective concentrations may be attributed to variations in experimental design, microbial strains, and antimicrobial testing conditions. Nevertheless, both studies consistently support the antimicrobial potential of *B. utriformis*, highlighting this species as a promising source of bioactive compounds with antibacterial activity.

In the study conducted by Asgharpour et al. [61], the hexane extract of *L. pyriforme* exhibited a highly selective antibacterial profile, showing inhibitory activity only against *S. aureus* ATCC 25923 with a MIC value of 125 μg/mL, while no detectable activity was observed against *E. coli*, *P. aeruginosa*, or *B. subtilis*. This narrow spectrum of activity was attributed to the apolar nature of the hexane solvent, which predominantly extracts lipophilic compounds such as sterols and fatty acid derivatives. In comparison, the hexane extracts evaluated in the present study also demonstrated limited and strain-dependent antimicrobial activity, supporting the notion that hexane is generally less effective than polar solvents for extracting broad-spectrum antimicrobial compounds from mushrooms. While selective inhibition was observed against certain bacterial strains in our study, the overall antimicrobial potency of hexane extracts was lower than that of methanol extracts and comparable to the restricted activity reported for *L. pyriforme.*

The antimicrobial activity observed in the present study can be partially attributed to the presence of phenolic compounds identified by GC–MS analysis, including substituted phenols such as 3,5-bis(1,1-dimethylethyl)phenol, 2,6-Bis(1,1-dimethylethyl)-4-(methoxymethyl)phenol and related derivatives. Phenolic compounds are well known for their ability to disrupt microbial cell membranes and inhibit essential enzymatic processes. In addition, the detection of fatty acid methyl esters, such as hexadecanoic, octadecanoic, and octadecadienoic acid methyl esters, may further contribute to the antimicrobial effect through membrane-targeting mechanisms, suggesting a synergistic role of phenolic and lipophilic constituents.

## 3. Materials and Methods

### 3.1. Chemicals and Reagents

Methanol, potassium persulfate, Trolox ((±)-6-hydroxy-2,5,7,8-tetramethylchromane-2-carboxylic acid), Cu(II) chloride, DPPH (2,2-diphenyl-1-picrylhydrazyl), potassium ferri-cyanide, trichloroacetic acid, ABTS (2-azino-bis-3-ethylbenzothiozoline-6-sulfonic acid), disodium hydrogen phosphate, and sodium sulfate anhydrous were purchased from Sigma Aldrich (St. Louis, MO, USA); Folin–Ciocalteu’s phenol reagent, sodium dihydrogen phosphate, ammonium acetate, ascorbic acid, ferric chloride, neocuproine and HCl were purchased from Merck (Damstadt, Germany); sodium carbonate was purchased from Tekkim Lab (Bursa, Türkiye); and hexane was purchased from Supelco (Sigma-Aldrich, St. Louis, MO, USA). All the chemicals and reagents used in the present study were of analytical grade.

### 3.2. Fungal Material

The macrofungi samples comprising the material for this study were collected at appropriate intervals from various locations where ecological conditions were suitable for fungal growth. Information about the locations (region, coordinates, and altitude) where the samples were collected and the photographs of the samples are provided in Table 1 and Figure 1.

The samples were identified based on the morphological characteristics such as color, size, number and shape of the cap, stipe, gill, basidiocarp, gleba, ascocarp and spores according to the current literature [32,33,34]. The samples were then air dried below 40 °C using a commercial vegetable drier, until the samples reached a constant weight, and kept in zipper bags away from direct sunlight in the laboratory. A further identification based on the isolation, amplification and sequencing of the internal transcribed spacer (ITS) region of the rDNA was also performed.

In this context, the DNA extraction of the samples was performed using a Macharey Nagel Plant II DNA extraction kit (Düren, Germany) according to the manufacturer’s instructions. The ITS region of the DNA was amplified by PCR using universal ITS-1 (5′-TCCGTAGGTGAACCTGCGG-3′) and ITS-4 (5′-TCCTCCGCTTATTGATATGC-3′) primers [62]. The obtained PCR products were sent to BM Labosis (Ankara, Türkiye) for sequence analysis. The data was compared with the NCBI GenBank database via BLAST for identification of the samples.

### 3.3. Extract Preparation

For preparing the extracts, 2 g of dried mushroom material, previously ground into a fine powder using a porcelain mortar and pestle, was mixed with 30 mL of methanol and homogenized at 9000 rpm for 5 min using a homogenizer (HG-15D, Daihan Scientific, Gangwon-do, Korea). The homogenate was incubated overnight at room temperature in the dark, followed by sonication for 40 min at 35 °C in an ultrasonic bath (Sonorex, Bandelin, Berlin, Germany). The resulting extract was filtered through Whatman No. 1 filter paper and subsequently concentrated to dryness using a rotary evaporator (HeiVap Value, Heidolph, Berlin, Germany). The obtained residue was re-dissolved in methanol to prepare a stock extract at a concentration of 200 mg/mL. These extracts were used to determine the total phenolic content and antioxidant properties of the fungal samples. The same extraction procedure was also applied using hexane as the solvent. Both methanol and hexane extracts were reconstituted in 5% dimethyl sulfoxide (DMSO) and used for antimicrobial activity assays.

### 3.4. Total Phenolic Compounds

The total phenolic compound contents of the methanol extracts of the fungal samples were determined using the Folin–Ciocalteu reagent method [63]. A total of 100 µL was taken from the extracts with a concentration of 100 mg/mL, and 1 mL of Folin–Ciocalteu’s phenol reagent (10-fold diluted) was added to it. After incubation at room temperature for 5 min, 1 mL of 7.5% Na_2_CO_3_ solution was added to the mixture and mixed with a vortex. The mixture was incubated again at room temperature in the dark for 90 min, and the absorbance of the resulting blue color was measured at 760 nm using a spectrophotometer (MultiSkan GO, Thermo Fisher Scientific, Waltham, MA, USA). The results obtained were calculated according to the gallic acid standard and presented as mg GAE/g dry weight.

### 3.5. Antioxidant Assays

#### 3.5.1. DPPH Radical Scavenging Activity

One of the commonly used methods for determining the free radical scavenging activity of mushroom extracts is the DPPH (2,2-diphenyl-1-picrylhydrazyl) scavenging activity method [64]. To generate a DPPH scavenging pattern, a five-step dilution series was prepared from 100 mg/mL methanol extracts of fungal samples. Briefly, 100 µL of each dilution was added to 2.9 mL of a 0.1 mM DPPH solution prepared with methanol. The mixture was vortexed and incubated at room temperature for 30 min. The absorbance of the resulting color was measured at 517 nm using a spectrophotometer. The DPPH scavenging activities of the fungal extracts were calculated according to the following formula:

%DPPH = [(A0 − Asample)/A0] × 100 (1)

where %DPPH is DPPH scavenging activity, A0 is the absorbance value of the blank tube at 517 nm wavelength, and Asample is the absorbance value of the tube containing the fungal extract at 517 nm wavelength. The DPPH radical scavenging activities of the mushroom extracts were calculated using the Trolox standard, and the results are presented as mmol TE/g dry weight.

#### 3.5.2. ABTS^+^ Cation Removal Activity

2,2-Azino-bis-(3-ethylbenzothiazoline-6-sulfonic) acid (ABTS) is another stable radical commonly used to measure the radical inhibition capacity of samples with antioxidant properties. For the ABTS assay, a mixture of 7.5 mM ABTS and 2.45 mM potassium persulfate (K_2_S_2_O_8_) solutions was prepared and incubated in the dark for 12–16 h to form the ABTS+ reaction solution. This solution was diluted to reach an absorbance of 0.700 ± 0.02 at 734 nm to obtain the ABTS^+^ working solution. In a 96-well plate, 10 µL of extracts or Trolox solutions at various concentrations was added to 200 µL of ABTS^+^ working solution, thoroughly mixed, and incubated in the dark for 6 min. The absorbance of the plate was measured at 734 nm, and the ABTS inhibition percentages of the samples were calculated using the following formula:

%ABTS = [(A0 − Asample)/A0] × 100 (2)

where %ABTS is ABTS scavenging activity, A0 is the absorbance of the ABTS^+^ working solution at 734 nm, and Asample is the absorbance of the sample at 734 nm [65,66]. A standard curve was established using the inhibition values of the Trolox solutions, and the results were expressed as mmol TE/g dry weight.

#### 3.5.3. Ferric Reducing Antioxidant Power (FRAP) Assay

The increase in absorbance of ferric ferrocyanide, a blue-colored complex exhibiting maximum absorbance at 700 nm, can be associated with the antioxidant activity of the sample, which reacts with potassium ferricyanide (K_3_[Fe(CN)_6_]) to form potassium ferrocyanide (K_4_[Fe(CN)_6_]). For the ferric reducing antioxidant power (FRAP) test, 30 µL of 0.2 M PBS at pH 6.6, 10 µL of extract or standard ascorbic acid solution at various concentrations, and 30 µL of 1% potassium ferricyanide (K_3_[Fe(CN)_6_]) solution were mixed and incubated at 50 °C for 20 min. After incubation, 30 µL of 10% trichloroacetic acid solution was added to the wells and mixed. The absorbance measurement of the plate was performed at 700 nm after adding 100 µL of distilled water and 20 µL of iron (III) chloride (FeCl_3_) [67]. A standard curve was created using ascorbic acid solution values, and the results were expressed as mmol AAE/g dry weight.

#### 3.5.4. Cupric Reducing Antioxidant Capacity (CUPRAC) Assay

The color change from bright yellow to orange in the presence of compounds acting as electron donors in the neocuproine and Cu(II)Cl_2_ mixture indicates the antioxidant capacity of a sample by reducing cupric (Cu^2+^) ions to cuprous (Cu^+^) ions. The CUPRAC assay was performed according to the method originally reported by Apak et al. [68]. A total of 50 µL of 0.1 M ammonium acetate buffer (pH 7.0), 50 µL of 10 mM Cu(II)Cl_2_, 50 µL of 7.5 mM neocuproine solution, 50 µL of distilled water, and 5 µL of extract or Trolox solution at different concentrations were added to a 96-well plate and incubated in the dark for 30 min. The absorbance of the samples was then measured at 450 nm using a spectrophotometer. A standard curve was created using the absorbance values of the Trolox solutions, and the results were expressed as mmol TE/g dry weight.

### 3.6. Chromatographic Analyses

#### 3.6.1. GC-MS

The chemical composition of the fungal samples was determined using Gas Chromatography–Mass Spectrometry (GC-MS). Forty milliliters of hexane was added to 1 g of dried and powdered fungal sample, homogenized at 8000 rpm for 5 min, and then tightly sealed and left to incubate for 24 h. The homogenate was then incubated in an ultrasonic bath at 40 °C for 30 min and centrifuged at 4000 rpm for 5 min. The extract was filtered through Whatman No. 1 filter paper, and the solvent was removed using a rotary evaporator. The resulting residue was resuspended in 5 mL of hexane. Then, 2 M KOH in methanol and 1 N HC1 solutions were used for saponification of the extracts (IUPAC Method 2.301). After phase separation, the upper layer was dried with anhydrous Na_2_SO_4_ and passed through a 0.45 μm nylon filter before injection into the GC-MS.

The composition of the hexane extracts was determined using a Shimadzu QP2010 Ultra GC-MS system equipped with a Restek Rxi 5MS column (30 m x 0.25 mm ID x 0.25 μm df). The injection block temperature was set to 240 °C. A 1 μL sample was injected into the system in split mode at a 1:40 ratio using an AOC2.0i autosampler. Helium was used as the carrier gas at a constant flow rate of 1.1 mL/min. The oven temperature program was set as follows: initial temperature 40 °C for 1 min, heated to 160 °C at a rate of 5 °C/min and held for 3 min, and finally heated to 250 °C at a rate of 5 °C/min and held for 11 min. The total analysis time was 57 min. The interface and ion source temperatures were 270 and 200 °C, respectively. All spectra were obtained in electron impact (EI) mode at 70 eV, and the mass range was set to 40–650 amu in full scan mode. The Wiley mass spectrum library (W9N11) and the Flavour and Fragrance Natural and Synthetic Compounds library (FFNSC 1.2) were used to identify the components detected by MS [69].

#### 3.6.2. LC–MS/MS

The phenolic profiles of the methanolic extracts obtained from the fungal samples were analyzed using a LC–MS/MS (Shimadzu 8040 system, Kyoto, Japan) equipped with an Inertsil ODS4 column (2.1 mm × 50 mm, 3 μm particle size; GL Science, Tokyo, Japan), maintained at 40 °C. Chromatographic separation of phenolic constituents was performed by injecting 10 µL of the sample and applying a gradient elution program with solvent A (1% formic acid in distilled water) and solvent B (1% formic acid in methanol) at a constant flow rate of 0.4 mL/min. The gradient conditions were set as follows: 95% A and 5% B at 0 min, gradually reaching 5% A and 95% B at 7 min. The total run time for each analysis was 12 min. Identification of individual phenolic compounds was achieved by comparing their retention times with those of authentic standards and by monitoring precursor and product ions in both positive and negative electrospray ionization modes. The operating parameters of the mass spectrometer were set as follows: an interface temperature of 350 °C, a desolvation line (DL) temperature of 250 °C, and a heat block temperature of 400 °C. Nitrogen was used as both the nebulizing and drying gas, with flow rates of 3 L/min and 15 L/min, respectively [70].

### 3.7. Microbiological Analyses

#### 3.7.1. Microorganisms

In this study, a total of 14 bacterial strains were utilized, including 4 multidrug-resistant strains. Additionally, the antifungal activity of the plant sample was tested against the standard yeast Candida albicans DSMZ 1386. The bacteria and yeast strains were obtained from the Department of Biotechnology at Niğde Ömer Halisdemir University’s Faculty of Science (Niğde, Türkiye).

#### 3.7.2. Microdilution Method (MIC)

Microdilution is a widely used and reliable method for evaluating antimicrobial susceptibility. This technique is based on the preparation of serial two-fold dilutions of the tested antimicrobial agent, typically ranging from 1 to 200 mg/mL, in an appropriate liquid growth medium. The assay can be performed either in 96-well microtiter plates (microdilution), which allows the use of smaller volumes while enabling high-throughput analysis. The antimicrobial activities of methanol and hexane extracts obtained from *B. utriformis*, *A. arvensis*, *T. claveryi*, and *T. aestivum*—which were recovered with 5% dimethylsulfoxide—with a final concentration of 200 mg/mL were evaluated by determining the minimum inhibitory concentration (MIC) using the microdilution method, in accordance with minor modifications. Pathogen microorganisms were adjusted to a turbidity equivalent to the 0.5 McFarland standard (approximately 1 × 10^8^ CFU/mL) and subsequently diluted to obtain the working inoculum. Serial two-fold dilutions of the mushroom extracts were prepared in LB broth, and 100 µL of each extract dilution was dispensed into sterile 96-well microtiter plates. Subsequently, 50 µL of fresh LB broth and 50 µL of the standardized microbial inoculum were added to each well, resulting in a final volume of 200 µL per well. Wells containing LB broth supplemented with microbial inoculum but without mushroom extract served as the positive growth control, whereas wells containing LB broth alone were used as the negative control. The MIC was defined as the minimum concentration of extracts (methanol and hexane) obtained from the mushrooms required to inhibit visible bacterial growth after 24 h of incubation. The results were expressed as mg/mL based on the mean values of three independent experiments [71,72].

### 3.8. Statistical Analysis

Group mean comparisons were performed using one-way ANOVA followed by Duncan’s multiple range test, considering *p* < 0.05 as statistically significant. Statistical analyses were carried out using IBM SPSS Statistics version 24.0 (SPSS Inc., Chicago, IL, USA).

## 4. Conclusions

The present study demonstrates that wild edible macrofungi represent promising natural sources of bioactive compounds with significant antimicrobial and antioxidant potential. Among the investigated species, methanol extracts consistently showed superior antimicrobial activity compared to hexane extracts, emphasizing the key role of polar bioactive constituents, particularly phenolic compounds. *B. utriformis* and *A. arvensis* exhibited the highest total phenolic contents and antioxidant capacities, while *T. claveryi* showed notable antimicrobial efficacy, including activity against selected multidrug-resistant bacterial strains. Chemical profiling of the investigated fungal samples using LC–MS/MS and GC–MS revealed that all four species were characterized by phenolic compounds including catechin, cinnamic acid and caffeic acid, and lipid profiles rich in unsaturated fatty acid methyl esters, especially linoleic and oleic acids, which may contribute synergistically to their biological activities. The strong correlation observed between phenolic content and antioxidant assays further supports the contribution of phenolic compounds to the overall bioactive potential of these fungi. Collectively, these findings underline the nutritional and pharmacological relevance of wild edible macrofungi and suggest their potential use as natural antioxidant and antimicrobial agents. Future studies focusing on compound isolation, mechanism of action, and in vivo evaluations are warranted to further explore their applicability in the food and pharmaceutical industries.

## Figures and Tables

**Table 1:** The collection dates, geographical positions, and GenBank accession numbers of macrofungi samples collected during the field studies.

Sample Name	Locations	Coordinates	Average Altitude	Collection Date	Accession No.
*Tuber aestivum*	Vize/Kırklareli	41°33′54″ N 27°43′09″ E	170 m	June, 2022	PX912447
*Terfezia claveryi*	Mazıdağı/Mardin	37°31′05″ N 40°26′32″ E	950 m	June, 2023	PX925061
*Agaricus arvensis*	Özyurt/Niğde	37°57′41″ N 34°51′24″ E	1870 m	May, 2022	PX907866
*Bovistella utriformis*	Çamardı/Niğde	37°54′55″ N 34°53′32″ E	1750 m	May, 2022	PX907867

**Table 2:** The content of total phenolic compounds and antioxidant activities of fungal extracts with 100 mg/mL concentration.

Sample	TPC	DPPH	ABTS	FRAP	CUPRAC
*T. aestivum*	18.52 ± 1.06 c	2.38 ± 0.15 b	1.46 ± 0.06 c	5.34 ± 0.08 c	3.91 ± 0.01 c
*T. claveryi*	16.66 ± 0.59 d	2.01 ± 0.23 c	1.30 ± 0.02 d	3.41 ± 0.05 d	2.57 ± 0.04 d
*A. arvensis*	27.14 ± 0.59 b	4.92 ± 0.01 a	1.90 ± 0.05 a	11.16 ± 0.27 a	8.04 ± 0.18 b
*B. utriformis*	29.61 ± 0.6 a	4.93 ± 0.04 a	1.66 ± 0.02 b	7.30 ± 0.14 b	10.45 ± 0.39 a

TPC: total phenolic compounds (mg GAE/g DW); DPPH: DPPH scavenging activity (mmol TE/g DW); ABTS: ABTS^+^ cation removal activity (mmol TE/g DW); FRAP: ferric reducing antioxidant power (mmol AAE/g DW); CUPRAC: cupric reducing antioxidant capacity (mmol TE/g DW). All data shown in the table is presented as the mean ± SE of three independent measurements. Different lowercase letters in each column indicate a significant difference at *p* < 0.05.

**Table 3:** Content of phenolic compounds in four macrofungi by LC–MS/MS (μg/g DW).

Compound	RT (min)	Precursor (*m*/*z*)	Product (*m*/*z*)	Ionization Mode	Content of Selected Phenolics (μg/g DW)			
					*T. aestivum*	*T. claveryi*	*A. arvensis*	*B. utriformis*
Catechin	2.279	291.1	138.9; 122.9	[M + H]^+^	14.724	3.268	16.359	18.657
Cinnamic acid	3.78	149.1	130.9; 103.2	[M + H]^+^	-	-	65.178	69.981
Caffeic acid	2.649	178.9	135; 134; 89	[M − H]^−^	0.952	0.804	0.033	0.027
2-5 dihydroxybenzoic acid	2.399	153.1	107.9; 109	[M − H]^−^	0.041	-	-	-
Trans-ferulic acid	2.946	192.8	132.9; 178	[M − H]^−^	0.098	0.061	0.047	0.040
Myrcetin	3.356	316.8	178.9; 151; 137	[M − H]^−^	-	-	-	-
Naringenin	3.608	270.8	150.9; 118.9; 92.9	[M − H]^−^	-	-	-	-
Quercetin	3.628	300.8	150.8; 121.4; 106.9	[M − H]^−^	0.013	0.011	-	-
Luteolin	3.737	284.8	217; 198.8; 174.9	[M − H]^−^	0.002	-	-	-
Chrysin	4.346	252.8	142.9; 119; 209.1; 106.9	[M − H]^−^	-	-	-	-
Tannic acid	2.18	182.9	123.6; 78.2	[M − H]^−^	-	-	-	-
Ellagic acid	3.624	300.8	229.1; 257.1	[M − H]^−^	-	-	-	-

RT: retention time; “-”: not detected. [M + H]^+^: The protonated molecular ion formed by the addition of a proton to the molecule in positive electrospray ionization (ESI) mode.

**Table 4:** Chemical composition of fungal extracts determined by GC-MS.

Compound	CF	MW	RT	RI	CT	Relative Concentration (%)			
						*T. aestivum*	*T. claveryi*	*A. arvensis*	*B. utriformis*
3-Hexanol	C_6_H_14_O	102	5.123	780	Alcohol	0.76	0.31	0.71	-
3,7-Dimethyldecane	C_12_H_26_	170	12.814	1086	Alkane	0.59	0.32	0.75	-
1-Iodononane	C_9_H_19_I	254	12.826	1330	Alkane	-	-	-	0.75
2,3,6,7-Tetramethyloctane	C_12_H_26_	170	14.249	958	Alkane	-	-	0.43	-
Dodecane	C_12_H_26_	170	17.26	1214	Alkane	0.74	0.43	0.55	0.54
5-Methyltetradecane	C_15_H_32_	212	19.177	1448	Alkane	0.68	-	-	0.74
4,6-Dimethyldodecane	C_14_H_30_	198	19.182	1285	Alkane	-	1.3	2.84	-
5-Butylnonane	C_13_H_28_	184	19.662	1249	Alkane	1.53	-	0.92	1.01
Hexadecane	C_16_H_34_	226	19.665	1612	Alkane	-	-	-	2.05
1-Ethyl-2-propylcyclohexane	C_11_H_22_	154	20.431	1140	Cycloalkane	0.5	-	-	-
1-Tridecanol	C_13_H_28_O	200	20.661	1556	Alcohol	0.5	0.29	-	1.41
7-Methyl-1-undecene	C_12_H_24_	168	20.668	1140	Alkene	-	-	0.52	-
Tetradecane	C_14_H_30_	198	23.018	1413	Alkane	3.96	2.45	3.41	3.08
2,6,10,14-Tetramethylpentadecane	C_19_H_40_	268	25.021	1707	Alkane	1.2	-	-	-
2,6-di-t-butyl-4-methylene-2,5-cyclohexadiene-1-one	C_15_H_22_O	218	25.285	1582	Ketone	3.35	1.75	4.02	3.3
Heptadecane	C_17_H_36_	240	25.617	1711	Alkane	3.84	0.58	3.93	1.51
7-Propyltridecane	C_16_H_34_	226	25.697	1548	Alkane	-	-	-	1.34
Octadecane	C_18_H_38_	254	25.788	1800	Alkane	-	-	1.36	2.99
3,5-bis(1,1-Dimethylethyl) phenol	C_14_H_22_O	206	26.113	1555	Phenol	0.35	0.37	-	1.51
2-Hexyl-1-octanol	C_14_H_30_O	214	26.745	1591	Alcohol	-	-	1.81	-
2-Hexyl-1-decanol	C_16_H_34_O	242	27.645	1790	Alcohol	0.75	-	0.49	1.01
Hexadecane	C_16_H_34_	226	28.902	1612	Alkane	2.83	3.14	3.05	4.16
2,6-Bis(1,1-dimethylethyl)-4-(methoxymethyl)phenol	C_16_H_26_O_2_	250	31.963	1803	Phenol	-	0.81	1.09	2.08
Tridecanoic acid, 4,8,12-trimethyl-, methyl ester	C_17_H_34_O_2_	270	32.749	1686	Fatty acid ester	-	-	0.77	-
Tridecanoic acid, 12-methyl-, methyl ester	C_15_H_30_O_2_	242	32.76	1615	Fatty acid ester	-	0.47	-	-
Octadecane	C_18_H_38_	254	34.802	1810	Alkane	5.26	3.01	3.16	4.99
9-Hexadecenoic acid, methyl ester	C_17_H_32_O_2_	268	37.429	1886	Fatty acid ester	-	0.57	-	-
Hexadecanoic acid, methyl ester	C_17_H_34_O	270	37.916	1878	Fatty acid ester	11.55	26.58	14.98	12.72
Benzenepropanoic acid, 3,5-bis(1,1-dimethylethyl)-4-hydroxy-, methyl ester	C_18_H_28_O_3_	292	38.403	2134	Ester	0.78	0.6	1.39	1.45
Nonadecane	C_19_H_40_	268	39.574	1900	Alkane	1.49	0.92	1.81	1.44
1-Octadecanol	C_18_H_38_O	270	41.36	2053	Alcohol	-	-	0.51	0.88
9,12-Octadecadienoic acid, methyl ester	C_19_H_34_O_2_	294	41.622	2093	Fatty acid ester	19.9	17.07	18.08	12.1
9-Octadecenoic acid, methyl ester	C_19_H_36_O_2_	296	41.737	2085	Fatty acid ester	12.69	11.54	1.34	1.11
Octadecanoic acid, methyl ester	C_19_H_38_O_2_	298	42.246	2077	Fatty acid ester	17.48	17.52	22.78	26.18
Pentadecanal	C_15_H_30_O	226	42.407	1701	Aldehyde	0.64	-	-	-
Docosane	C_22_H_46_	310	43.299	2200	Alkane	2.36	1.23	1.7	2.69
2-Propenoic acid, pentadecyl ester	C_18_H_34_O_2_	282	45.468	1968	Ester	1.62	1.3	1.44	1.22
Cyclopentanetridecanoic acid, methyl ester	C_19_H_36_O_2_	296	46.034	2120	Fatty acid ester	0.33	-	-	-
Eicosanoic acid, methyl ester	C_21_H_42_O_2_	326	46.045	2276	Fatty acid ester	-	0.94	1.01	-
Hexadeca-2,4-dienoic acid, methyl ester	C_17_H_30_O_2_	266	46.322	1875	Fatty acid ester	-	0.47	-	-
6,9,12,15-Docosatetraenoic acid, methyl ester	C_23_H_38_O_2_	346	47.088	2507	Fatty acid ester	-	0.63	-	-
2,6,11,15-Tetramethylhexadecane	C_20_H_42_	282	47.357	1753	Alkane	-	-	0.69	-
Tetracosane	C_24_H_50_	338	47.363	2400	Alkane	1.27	0.77	-	2.52
9,12-Octadecadienoic acid, ethyl ester	C_20_H_36_O_2_	308	48.998	2193	Fatty acid ester	-	0.47	-	-
Docosanoic acid, methyl ester	C_23_H_46_O_2_	354	50.305	2475	Fatty acid ester	-	0.88	-	-

RT: retention time (min); RI: Kovats’ retention index; CF: chemical formula; MW: molecular weight; CT: compound type.

**Table 5:** Minimum inhibitory concentrations (MICs) of methanol extracts against bacterial and yeast strains (mg/mL).

Strains	MIC (mg/mL)			
	*T. aestivum*	*T. claveryi*	*A. arvensis*	*B. utriformis*
*Enterococcus faecalis* ATCC 29212	0.8	0.8	1.6	0.8
*Bacillus subtilis* DSMZ 1971	1.6	3.125	25	50
*Staphylococcus aureus* ATCC 25923	200	100	100	50
*Pseudomonas aeruginosa* DSMZ 50071	200	100	100	100
*Escherichia coli* ATCC 25922	200	100	50	100
*Staphylococcus epidermidis* DSMZ 20044	200	200	50	25
*Enterobacter aerogenes* ATCC 13048	100	50	100	50
*Microccoccus luteus* M41	25	25	100	12.5
*Escherichia coli* MDR	200	50	25	100
*Klebsiella pneumoniae* MDR	200	25	25	100
*Salmonella typimurium* SL1344	200	50	100	100
*Streptococcus pneumonia* MDR	25	50	12.5	25
*Staphylococcus aureus* MRSA	-	25	-	50
*Candida albicans* DSMZ 1386	6.25	3.125	200	100
*Bacillus cereus* RSKK 863	3.125	1.6	25	50

All MIC values are expressed in mg/mL. “-” indicates no inhibition was observed at the tested concentration range.

**Table 6:** Minimum inhibitory concentrations (MICs) of hexane extracts against bacterial and yeast strains (mg/mL).

Strains	MIC (mg/mL)			
	*T. aestivum*	*T. claveryi*	*A. arvensis*	*B. utriformis*
*Enterococcus faecalis* ATCC 29212	50	25	50	50
*Bacillus subtilis* DSMZ 1971	100	50	25	50
*Staphylococcus aureus* ATCC 25923	200	100	-	100
*Pseudomonas aeruginosa* DSMZ 50071	-	-	-	-
*Escherichia coli* ATCC 25922	-	-	-	-
*Staphylococcus epidermidis* DSMZ 20044	-	-	100	200
*Enterobacter aerogenes* ATCC 13048	-	-	-	-
*Microccoccus luteus* M41	50	50	50	100
*Escherichia coli* MDR	-	-	-	-
*Klebsiella pneumoniae* MDR	-	-	100	200
*Salmonella typimurium* SL1344	-	-	-	-
*Streptococcus pneumonia* MDR	-	-	-	-
*Staphylococcus aureus* MRSA	-	-	-	-
*Candida albicans* DSMZ 1386	-	-	-	-
*Bacillus cereus* RSKK 863	50	25	25	50

All MIC values are expressed in mg/mL. “-” indicates no inhibition was observed at the tested concentration range.

## Data Availability

The original contributions presented in the study are included in the article material; further inquiries can be directed to the corresponding author.

## Abbreviations

The following abbreviations are used in this manuscript:

ITS	Internal Transcribed Spacer
DPPH	2,2-diphenyl-1-picrylhydrazyl
ABTS	2-azino-bis-(3-ethylbenzothiozoline-6-sulphonic acid)
FRAP	Ferric Reducing Antioxidant Power
CUPRAC	Cupric Reducing Antioxidant Capacity
TPCs	Total Phenolic Compounds
LC-MS	Liquid Chromatography–Mass spectrometry
GC-MS	Gas Chromatography–Mass Spectrometry
MIC	Minimum Inhibitory Concentration
